# Tree-Weighting for Multi-Study Ensemble Learners

**Published:** 2020

**Authors:** Maya Ramchandran, Prasad Patil, Giovanni Parmigiani

**Affiliations:** 1 Department of Biostatistics, Harvard T.H. Chan School of Public Health; 2 Department of Data Sciences, Dana-Farber Cancer Institute, Boston, MA 02115, USA

**Keywords:** Ensemble learning, Random Forests, Replicability, Multi-study learning

## Abstract

Multi-study learning uses multiple training studies, separately trains classifiers on each, and forms an ensemble with weights rewarding members with better cross-study prediction ability. This article considers novel weighting approaches for constructing tree-based ensemble learners in this setting. Using Random Forests as a single-study learner, we compare weighting each forest to form the ensemble, to extracting the individual trees trained by each Random Forest and weighting them directly. We find that incorporating multiple layers of ensembling in the training process by weighting trees increases the robustness of the resulting predictor. Furthermore, we explore how ensembling weights correspond to tree structure, to shed light on the features that determine whether weighting trees directly is advantageous. Finally, we apply our approach to genomic datasets and show that weighting trees improves upon the basic multi-study learning paradigm.

## Introduction

With increasing availability of multiple datasets measuring the same outcome and many of the same features, it is important to consider the totality of this information to train replicable predictors. Training and validating on a single study can often produce inflated reports of accuracy, especially when compared with training and validating on different studies.^1^ This motivates the use of ensemble learning methods to combine predictions based on multiple studies. A cross-study learner (CSL)^2^ is an ensemble of predictors trained separately on different studies. Single Study Learners (SSL’s) refer to any algorithm that produces a prediction model using a single study. The CSL combines SSL’s using a specified weighting strategy, creating a single predictor that can be applied to external studies. For *K* training datasets, let ${\hat{Y}}_{k}(x)$ be the prediction from SSL*k* at a new point *x*, and by *w*_*k*_, for *k* = 1*, ..., K*, the weight given to SSL_*k*_ in the ensemble. The prediction made by the CSL is ${\hat{Y}}_{k}(x)=\sum_{k=1}^{K}w_{k}{\hat{Y}}_{k}(x).$ Ensembles may be created using multiple SSL types (e.g. a neural net and a support vector machine), or a single type, which will be the setting explored in this paper. Depending on the weighting scheme utilized, CSLs can enhance replicability and robustness to different feature-response relationships across studies, without explicitly modeling these differences.

Patil and Parmigiani^2^ have previously explored using multiple training studies to devise ensembling strategies. As a benchmark, they considered a single learner trained by merging all the studies (merging). They found that in the presence of cross-study heterogeneity, merging and meta-analysis performed worse than CSLs. They additionally observed that when training with Random Forests^3^ (RF) as the SSL, ensembling always produced a better predictor than merging, even in simulations with no between-study heterogeneity in the feature-outcome relationship. It would appear in this case that merging all studies, thereby including more data points with the same feature-outcome relationship in training, would be most likely to result in a prediction rule that closely reflects the data generating mechanism. Patil and Parmigiani found this to be the case for all SSLs they considered except RF. For RF, ensembling SSLs proved better than merging, even though each dataset provides fewer samples for a single forest to learn the same prediction rule. This unexpected finding motivates our present exploration into the properties of Random Forest and optimal tree-weighting strategies.

RF^3^ uses bagging^4^ to train an ensemble of decision trees. In the original algorithm, every tree within the Forest is given an equal weight in determining a prediction. We consider RF as our SSL to explore ensembling a group of SSL’s that are themselves ensemble learners. We are particularly interested in the effect of extracting the trees trained by each SSL forest and weighting them directly, instead of simply assigning weights to each forest and giving all internal trees equal weighting. We consider weighting approaches that reward cross-study replicability within the training set. Rewarding cross-study replicability on the tree level should increase the robustness of the overall ensemble to differing population parameters, such as between-study heterogeneity in the relationships between the features and the outcome.

Ours is the first tree-weighting scheme designed for the multi-study setting. Several approaches exist for weighting trees within random forests, include using a weighted random sampling scheme to train the trees,^5^ weighting the trees themselves^6,7^ or some combination of the two^8^.^9^ Little has been developed on regression tree-weighting strategies, which is the primary focus of this paper. Winham et. al.^6,^ weighted Random Forests (wRF) uses tree-level weights to upweight more accurate trees as evaluated on a held-out test set. The authors showed that while wRF can outperform RF in high-dimensional data, this improvement only holds for larger effect sizes than typically seen in real data. We propose a tree-weighting method that improves on the existing paradigms given realistic effect sizes for multiple studies. In summary, this paper examines whether directly weighting trees improves on weighting forests, using simulations based on data including gene expression measurements and survival outcomes in ovarian cancer patients. We additionally apply our tree-based ensembling methods to other genomic multi-study and large-scale datasets

## Methods

### Ovarian Cancer Datasets.

CuratedOvarianData^10^ from Bioconductor in R provides data for gene expression meta-analysis of patients with ovarian cancer. In this study, we use all 15 studies in CuratedOvarianData that provide survival information without any missing data in the features. Sample sizes range from 42 to 510 subjects. We focus on 2,909 gene features observed in all studies. The gene expression features in each dataset are normalized.^10^

### Simulations.

We used feature vectors resampled from CuratedOvarianData, to ensure realistic distributions and covariances. For N = 100 iterations for each set of generative parameters, we randomly separated the 15 datasets from CuratedOvarianData into *K* = 10 training and *V* = 5 validation sets. Per iteration, we reduced each dataset to the same randomly sampled 100 genes out of the 2909 available. We chose 10 of these 100 genes randomly to create a linear data-generating model and drew their coefficients uniformly from [*−*5*, −*0.5] ∪ [0.5, 5], so each has a non-zero contribution to the outcome. We added user-specified between-study heterogeneity in the feature-outcome relationship through coefficient perturbation. We chose perturbation windows to explore both small- and large-scale between-study heterogeneity, in line with the differences in feature-outcome relationships we observe in the actual data. If **c** is the vector of coefficients and *l* the desired level of heterogeneity, we drew uniformly from [**c** − *l,* **c** + *l*]. In every simulation, we assigned 5 of the training datasets low levels of coefficient perturbation, the other 5 higher levels, and the validation sets an intermediate level, to provide more pronounced variation in training than in testing. At baseline, we set *l*’s as .25, 1, and .4 respectively, to introduce relatively small perturbations throughout, with the validation level representing a middle ground between the two levels used in training. We varied the heterogeneity levels throughout the analysis, to evaluate the performance of the ensembles; in all figures, the heterogeneity level corresponds to the highest level in the training sets. The low level for the other 5 training sets was held constant at .25, and the intermediate level given to the validation sets was half of the highest level. We used a location shift instead of a scale change so that the features could be affected differently by between-dataset variation. We then generated the outcome vector *Y*_*k*_ for each study *k* = 1*, ...*15 conditional on the chosen subset of the observed predictors and the simulated coefficients. We also performed simulations with interactions between some of the features. We considered two scenarios: (1) Two datasets in the training set and two in the validation set contain interaction terms, and (2) Six datasets in the training set and two in the validation set have interaction terms. For all scenarios involving interaction terms, we chose moderately low baseline heterogeneity parameters. Further modifications used in baseline simulations are described in the Results section.

### Stacked regression weights.

Our goal is to develop ensembles of RF SSL’s that improve predictions. To construct the weights for each SSL in the ensemble, we used the multi-study version of the stacked regression method,^2^ which rewards cross-study generalizability of SSL’s in determining the ensembling weights. Stacked regression^11^ forms linear combinations of multiple predictors that improves on the performance of any single one. The weights given to each predictor are determined by cross-validation and least squares regression. Breiman^11^ explored several modifications to least squares and found that imposing a non-negativity constraint to the coefficients gave the best results. We considered variations here, including Lasso and adding or eliminating an intercept, and found overall that using stacked regression with a Ridge constraint and intercept term produced the most robust ensemble in our setting. Ridge regression shrinks coefficients rather than directly zeroing some out; this appears to allow for greater generalizability of the resulting predictor. Setting the contributions of SSL’s directly to zero within training due to poor cross-validation performance may promote overfitting to the training datasets, as such SSL’s could provide value when faced with an observation arising from a new dataset with a potentially different data generating model.

We first trained an SSL on each training set. We then stacked the predictions into matrix $\text{T}=[{\hat{Y}}_{1}^{\prime },...,{\hat{Y}}_{K}^{\prime }]\prime ,$ where ${\hat{Y}}_{k}=[{\hat{Y}}_{1k}...{\hat{Y}}_{Kk}]\prime$ for *k* = 1*,..., K*; ${\hat{Y}}_{ik}$is the vector of predictions of SSL*_k_* on dataset *i*, and ${\hat{Y}}_{k}$ is the stacked vector of all predictions made by SSL_*k*_ on every training dataset. T is *N × K*, with *N* the total number of observations in all datasets. We stacked the true outcomes across all training studies into the *N ×* 1 vector *Y* . We then regressed *Y* against *T* , with a ridge penalization and non-negativity constraint. The SSL weights **w**_*stack*_ are determined by solving $\text{mi}{\text{n}}_{\text{W}}{}_{stack}\left\|{\left(Y-(\text{T}\times {\text{w}}_{stack})\right)}^{2}\right\|$ such that ${\text{w}}_{stack}>0\;\text{and}\;\left\|{\text{w}}_{stack}^{2}\right\|\;\leq \lambda ,$ where λ is optimized using the cross-validation procedure in the glmnet package in R.^12^

Throughout, *Weighting Trees* will indicate individually weighting each tree using stacked regression with Ridge regularization and a non-negativity constraint, while *Weighting Forests* will indicate using the same method on the predictions made by whole forests. The term *Unweighted* will refer to the ensemble created by giving every single-study forest equal weighting. The term *Merged* will refer to the predictor trained using a single RF on the dataset formed by merging the 15 studies. The difference between the Weighting Trees and Weighting Forests approaches is in what constitutes an SSL: individually weighting trees corresponds to first training a Random Forest on each study, then extracting the trees and treating each of the extracted trees as an SSL. If *m* is the number of trees per forest, the length of **w**_*stack*_ is *K × m* for the Weighting Trees approach and *K* for the Weighting Forests approach. In some analyses we compare tree-level weights for Weighting Trees to the tree-level weights implied by Weighting Forests, which we define by dividing each forest-level weight from **w***stack* by *m*, as each tree within the *k*^*th*^ forest is implicitly assigned weight *w*_*stack,k*_ */m*.

#### Number of trees per forest.

The RF implementation in the randomForest package in R uses 500 trees per forest as a default. In general, the performance of RF increases as a function of the number of trees before reaching a plateau; for this reason, the number of trees/forest is not typically considered to be a tuning parameter optimized through cross-validation. We found in our preliminary simulations that the order relationship between approaches was conserved as the number of trees per ensemble increased; this is illustrated in Figure S2 from the supplement. We also saw larger gains in predictive performance for the ensembling methods we evaluate when using greater numbers of trees per forest. For speed of computation, we used 10 trees per forest in all ensembles of our main analyses, so we anticipate that the improvements we report are conservative representations of what is possible with larger forests. We trained the Merged learner with the same number of trees contained in the total weighted ensembles, to keep the total number of trees for each method equal. Typically, this means that there are 100 trees per ensemble, unless otherwise specified.

## Simulation Results

### Goals.

Our simulations evaluate the overall behavior of different tree-weighting approaches in realistic scenarios. The presence of interactions between variables associated with the outcome in the true model is prevalent in real datasets, including those arising in genomics. Identifying such interactions is typically difficult, and the information that could be leveraged by recognizing the utility of these terms is often lost. We were therefore interested in how the presence of interactions and the magnitude of their coefficients would affect the structure of trees trained by RF and the performance of the ensembles. We also study differences in the relationship between the covariates and the outcome across studies, and evaluate how these affect cross-study generalizability of trees within the ensemble.

### Baseline analyses.

**Fig. 1:**
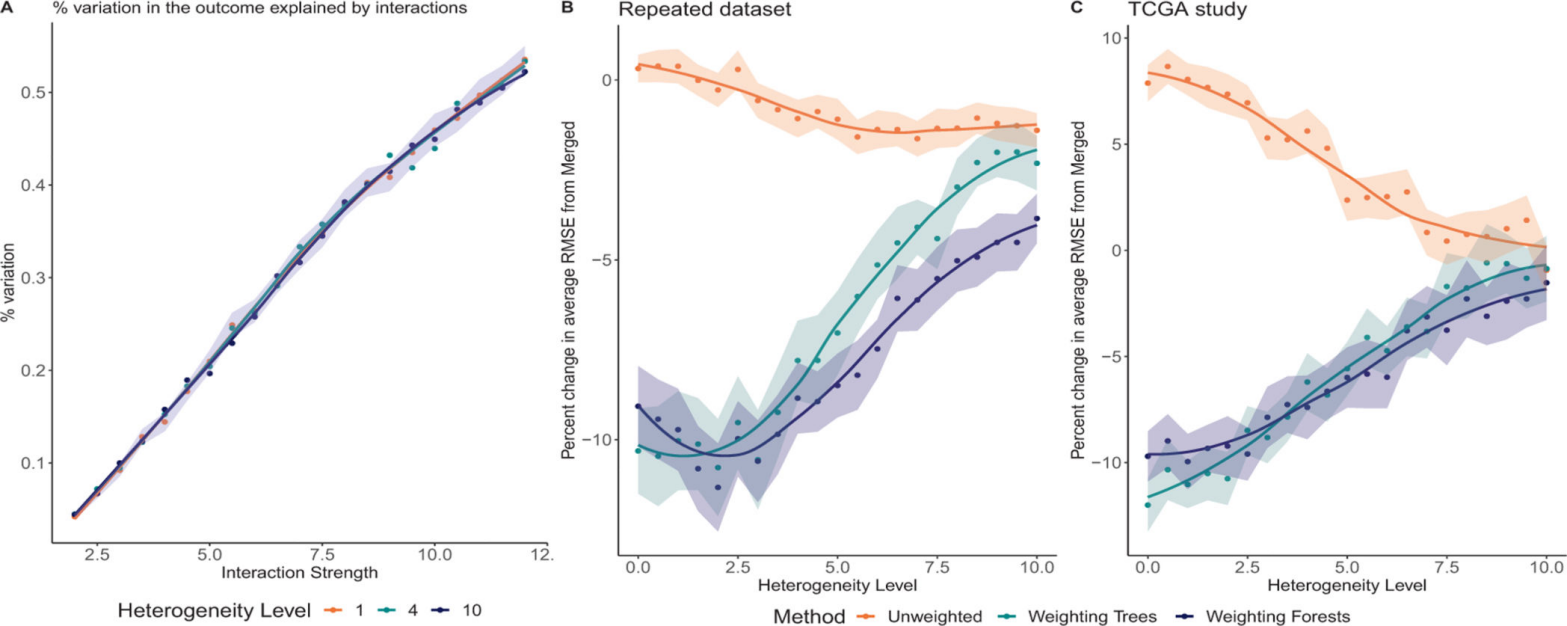
Baseline analyses. **(A)** Percent variation in outcome explained by interactions, as a function of magnitude of interaction coefficients in the generating model. **(B)**-**(C)** Percent change in average RMSE of each ensembling approach compared to the Merged learner, as a function of between- study heterogeneity level. In **(B)** all training sets have identical feature distributions while in **(C)** the TCGA study is randomly split into 5 sub-datasets at every iteration for training and testing. Weighting Trees and Weighting Forests significantly improve upon Merged, with the difference in performance decreasing as heterogeneity increases. Smoothing is applied to reduce simulation noise.

Throughout this section we report averages over 100 simulations per scenario, with 95% confidence bands computed as mean *±*1.96*×* standard error. We begin in Figure 1A by detailing the relationship between the size of interaction terms and the variation in the outcome, to get a sense of the importance of interactions, at different levels of heterogeneity. We observe a roughly linear relationship between interaction strength and percent variation. The level of between-study heterogeneity does not affect this conclusion.

We next examine the relationship between the level of between-study heterogeneity and the performance of the ensembling approaches. To interpret panels B and C of Figure 1, we must first distinguish between “feature distribution heterogeneity” and “feature effect heterogeneity” (see also^13^). The former refers to changes in the distribution of features between datasets. The latter refers to changes in the relationship between the features and the outcome: in our simulations, it is captured by changes in the coefficients of the generating linear model, and is used throughout as the horizontal scale in the figures. At baseline, we explored removing feature distribution heterogeneity and evaluating ensembles at differing levels of feature effect heterogeneity. Both types of heterogeneity may influence the utility of ensembling, so these simulations helped us more sharply analyze feature effect heterogeneity alone.

In the simulation used to generate Figure 1B, we set *K* = 10. Each of the *K* studies has the same sample size and the same feature set obtained by repeating a single randomly chosen ovarian cancer dataset *K* times, thus removing feature distribution heterogeneity. Each iteration has a different level of feature effect heterogeneity. In each we train the Merged learner and the SSL’s and evaluate them on 5 independent datasets not used in training. The Weighting Trees and Weighting Forests approaches perform better than the Merged learner across all levels of heterogeneity considered, while the Unweighted method generally only slightly improves on the Merged. Training an SSL on each set of observations and reweighting upwards (upweighting) learners with better cross-study validation performance (which at level 0 is akin to cross-validation) produces markedly better results. The difference is most pronounced at levels of feature effect heterogeneity around 2, where it exceeds 10%, and is substantial at level 0. As the level of heterogeneity rises, the RMSE’s increase, and the difference in performance among approaches decreases. We also evaluated the performance of a single learner trained on one copy of the dataset chosen to provide the features in the training set, and found that in the absence of feature distribution heterogeneity, the ensembles using the stacking weights trained on the repeated training set outperform this single learner.

The dataset used to train the Merged includes each sample in the training dataset 10 times, a somewhat artificial setting. Alternatively, for Figure 1C, in each iteration, we split the TCGA study within curatedOvarianData (comprising 510 subjects) into 5 equally sized pseudo-datasets. We used 4 to train the merged learner and SSL’s, and the 5th to test the resulting ensembles. This simulation helped us investigate whether differences in the the distribution between smaller sub-datasets and the whole dataset would be sufficient to result in a better performance of ensembling approaches compared to merging. Both the Weighting Trees and Weighting Forests approaches outperform the Merged at all heterogeneity levels, while the Unweighted ensemble results in a decrease in performance.

### Overall performance of ensembling approaches.

**Fig. 2:**
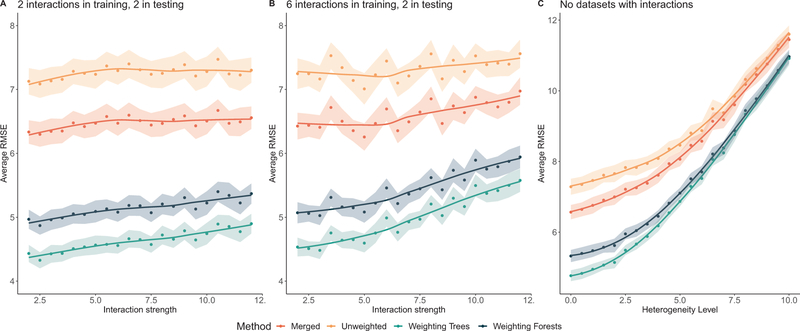
Average RMSE’s of ensembling approaches (color labeled) across different data-generating scenarios, as a function of increasing interaction strength or heterogeneity. **(A)** 2 datasets with interaction terms between features in the outcome-generating generating mechanism are included in the training set, and 2 are included in the testing set. **(B)** 6 datasets with interactions are included in the training set, 2 in the testing set. **(C)** No datasets with interaction terms are included in either training or testing, and performance is evaluated for increasing feature effect heterogeneity.

Our next simulations include varying levels of both feature distribution and feature effect heterogeneity, and additionally explore including interaction terms in the outcome-generating mechanism, representing the complexity we expect to see in real data. Figure 2 displays average RMSE’s over 100 iterations for the various ensembling approaches for three different scenarios. Out of 100 available features, 10 affect the continuous outcome. Panel A corresponds to interaction scenario (1) as described earlier, while panel B corresponds to scenario (2). Panel C includes no interactions and considers the effect of increasing the level of feature effect heterogeneity.

Overall, both Weighting Trees and Weighting Forests perform significantly better than either of the simpler approaches considered as benchmarks. Interestingly, ensembling forests is only better than merging when we suitably weight each forest or tree, since the Unweighted approach consistently performs worse than the Merged. The Merged and Unweighted each give the same overall weight to each tree (1/100), but they differ in the number of candidate data points available to form a bootstrap sample to train each tree. We can extrapolate that training each tree on the merged dataset produces a predictor with better generalizability than restricting each tree to a single dataset and using simple averaging to combine the resulting forests. This motivates the utility of using weighting approaches that reward cross-study replicability when constructing an ensemble of ensembles. Otherwise, it may be both more efficient and accurate to rely on the ensembling produced within a single RF.

Furthermore, the Weighting Trees strategy consistently performs better than Weighting Forests as well as the other baseline approaches. This follows the intuition that we can create a more robust ensemble by rewarding cross-study replicability at the individual tree level, by considering the strengths and weaknesses of individual trees rather than forests in terms of out-of-sample cross-study validation performance within the studies in the training collection. A comparison of panels A and B from Figure 2 indicates that scenarios with more training datasets containing interactions do not generally correspond to improved performance of any of the ensembling constructions considered; additionally, there is more variability around the average trend line. This suggests there is a balance in creating an optimally heterogeneous set of training studies for ensembling, and that the presence of heterogeneity could be more important than the sample size.

Figure 2C demonstrates that as the level of feature effect heterogeneity increases, the differences in performance of the ensembling approaches we considered decreases until they become virtually indistinguishable. At the lowest level of heterogeneity, we observe a significant separation between Weighting Trees, Weighting Forests and the simpler approaches. This suggests that in our data, there is intrinsic cross-study heterogeneity in the distribution of the features, that motivates utilizing replicability weights over merging or simple averaging, supporting the conclusions in the baseline simulations.

### Tree structure and variable importance measures.

One of our primary interests in this study was to explore how the trees trained by Random Forest capture relationships between the variables and the outcome that are stable across studies, to elucidate which features are important to weighting schemes that reward replicability. To this end, we examined average trends across several different components of the internal tree structures. Table 1 displays results about how tree structure and variable importance measures are related to tree weights. The ‘true’ variables are the variables chosen to linearly combine to generate the outcome in each simulation. All averages are taken over 100 simulations; 2 datasets in both the training and testing group contain interaction terms. For all ensembles, 9 variables per tree are available to create splits at each node; in the data generating mechanism, 3 variables out of the 10 associated with the outcome are involved in interaction terms.

For both tree-based and forest-based ensembles, trees with larger weights also have a higher percentage of true variables, number of variables involved in interactions, and true variables with higher variable importance. Interestingly, the frequency of true variables and true interaction variables in trees in the Merged learner is higher than in the top decile for either Weighting Forests or Weighting Trees. Yet, the Merged has worse overall performance than both ensembling approaches. We speculate that these metrics of tree-level variable importance are imperfect markers of cross-study generalizability, as they do not address the stability of a variable’s effect which is required for replicability. The true variables in each iteration of the simulation are shared across all studies, so the frequency at which such variables appear within trees is not intrinsically a study-specific feature. Similarly, the variables chosen to be involved in interaction terms are shared across all studies chosen to contain interactions, trees trained on such studies share similarities across studies. However, the coefficients do vary, and cross-study learners attempt to build predictors that are more robust to this variation.

The top decile of tree-based ensembles and forest-based ensembles contain on average nearly half of the 3 variables involved in interactions, with the tree-based average slightly higher than that for the forest-based ensemble. This is significantly higher than either the total average or the average within the lowest decile for either method, indicating that when the true relationship between the features involves interactions between variables, trees containing such variables have markedly increased cross-study generalizability and are therefore given higher weights within the ensemble.

### Distribution of tree-level weights.

**Fig. 3:**
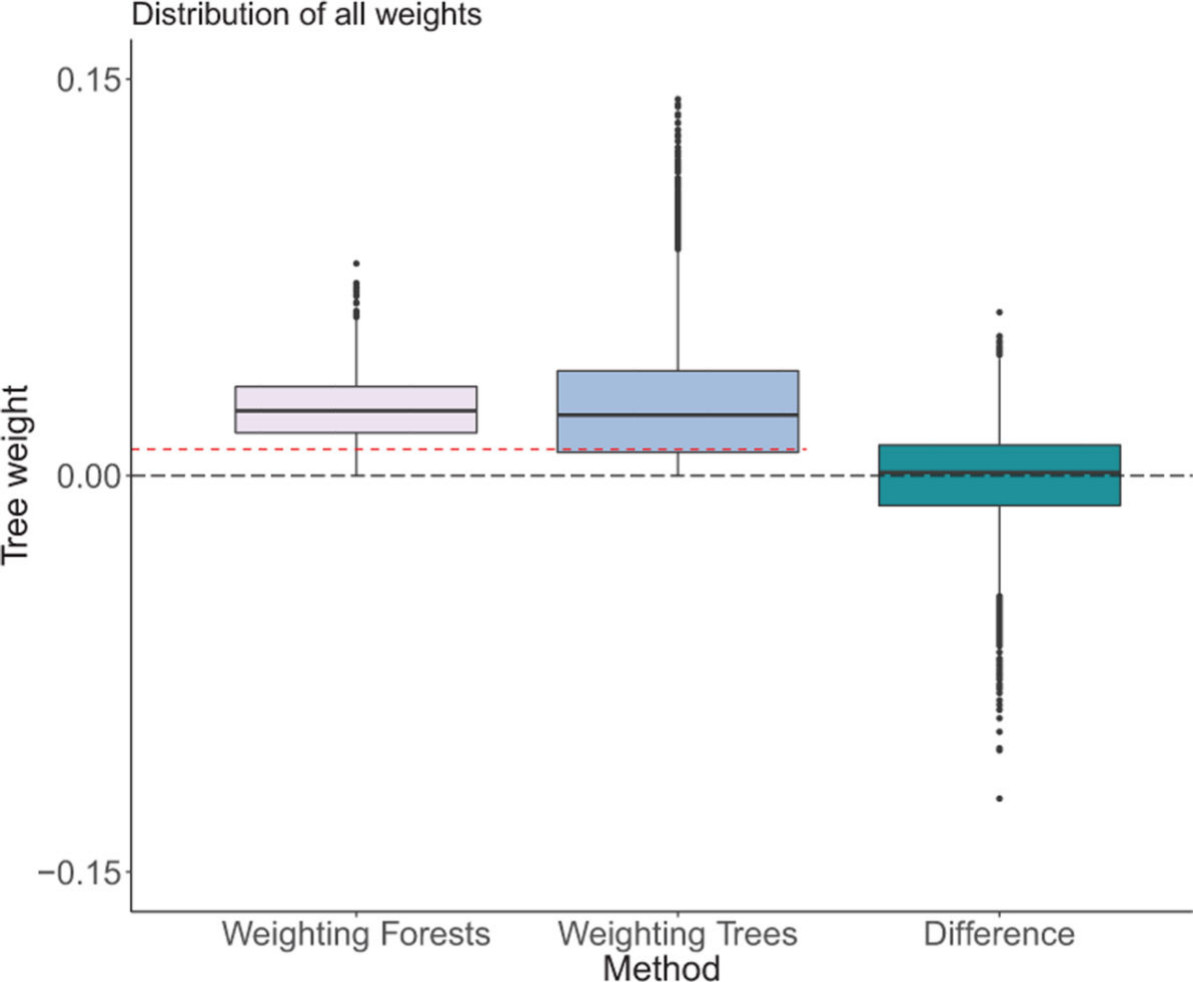
Distribution of tree-level weights using the Weighting Trees or Weighting Forests methods, as well as their difference. 2 training sets contain interaction terms. Tree-level weights for Weighting Forests are obtained by dividing the forest-level weight returned by the stacking algorithm by the number of trees per forest. Correspondingly, each point in Weighting Forests represents the value of the weight given to 10 trees. The dashed red line at *y* = .01 represents the weight given to every tree within the Merged and Unweighted.

In general, the distributions of the weights given to individual trees have a similar location, but are more dispersed than those given to forests, and have a more pronounced upper tail. From Table 1, we see that for the Weighting Trees approach, the lowest decile of trees have weights that are 10-fold lower compared to the merged, while the top decile is approximately 7 times higher. The Weighting Forests method has a smaller inter-decile range. Forest-based ensembles do not allow for control over tree-level weights, resulting in a narrower overall distribution. The benefits afforded by a few trees are potentially averaged out in the performance of the corresponding forest and potentially not rewarded optimally. Figure 3 illustrates this. We implement stacking without a normalization of weights. Both tree- and forest-weighting approaches produce distributions centered above 0.01, the weight that would be given to each tree if all were equally weighted, as in the Merged and Unweighted. The distribution of the difference between the weight given to the same tree by the Weighting Forests and Weighting Trees approaches is centered around zero, but displays a pronounced lower tail, suggesting that Weighting Trees may identify a relatively small subset of trees for up-weighting, while reducing the weight of most others, compared to Weighting Forests. Consistently, as displayed in Table 1, the top decile of the Weighting Forests distribution is on average smaller than that for Weighting Trees while the overall means are roughly equal. Essentially, trees deemed by the Weighted Trees approach to be among the least beneficial in the ensemble may still receive moderate or high weight if the whole forest is weighted together. This may lead to the decrease in performance we see throughout.

Several factors (both measured and unmeasured) contribute to improving cross-study generalizability of individual trees in addition to the presence of variables involved in interaction terms. It appears that the interplay between all such components contributes to determining the weight of each tree, and that isolating the presence or absence of any single one will not explain significant variation in weights.

## Data Application

To explore the performance of these classifiers on real data with natural feature-outcome relationships, we considered relatively high-dimensional multi-study datasets similar to those used in our simulations, in which there may plausibly be inter-study heterogeneity and interactions between features. Gene expression and clinical data were natural candidates.

We considered datasets in CuratedBreastData from Bioconductor in R, which provides gene expression and clinical data for 34 studies following patients with breast cancer.^14^ Clinical information is binary; five studies measured Overall Survival (OS), our clinical outcome of interest. OS is defined as 1 if the patient survives to the end of the study period, and 0 otherwise. Study periods may vary, resulting in a further source of heterogeneity, affecting feature effects. The sample sizes range from 14 to 118, with a total of 336 subjects across studies. The five datasets have 76 gene features in common and 47 clinical features with fewer than 10 missing datapoints across studies. These 123 variables were used as features in the analysis. We tested the performance of the ensembling approaches when predicting OS while training on four studies and validating on the fifth, using log loss as our metric of success. Since randomForest trains a different set of trees at each iteration, we replicated the method 100 times to obtain average log loss values and standard errors.

**Fig. 4:**
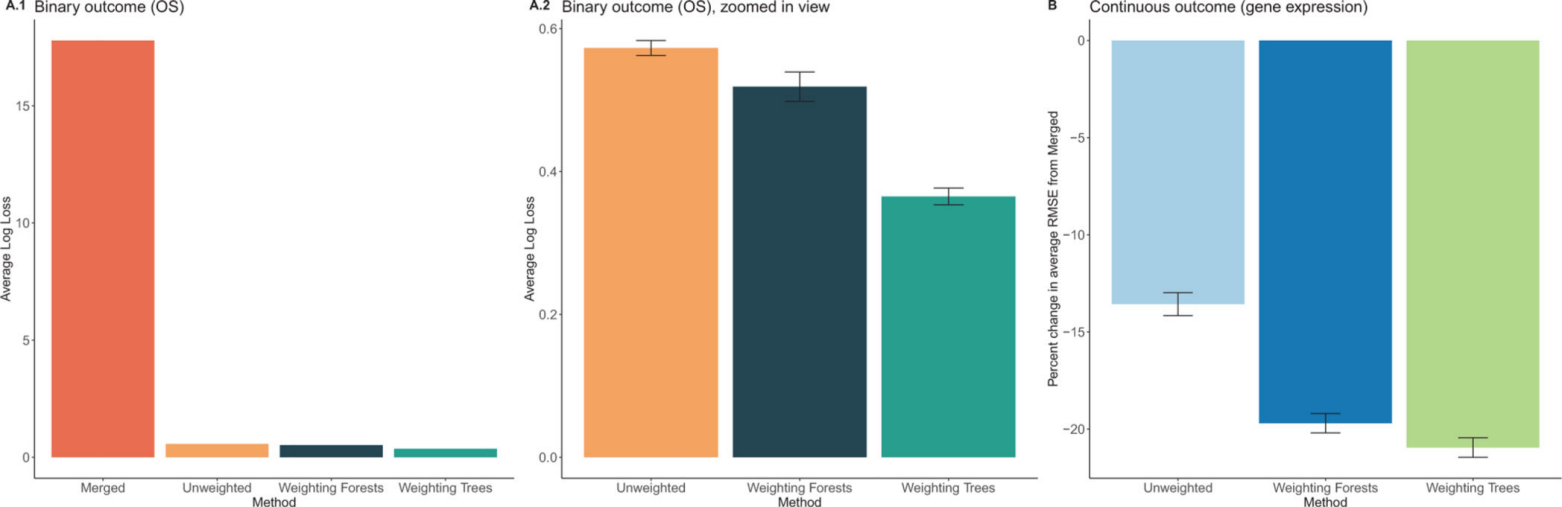
Performance of ensembling approaches on the breast cancer datasets in the multi-study setting, with associated 95% confidence intervals. **(A.1)** Average percent change in prediction Log Loss from the Merged for each of the ensembling approaches on the binary outcome variable, Overall Survival (OS). Confidence intervals were obtained by training each of the ensembling approaches 100 times, with differences in performance across iterations induced by the randomization within the Random Forest algorithm. **(A.2)** A view of panel A.1, without the Merged learner to improve scaling, so differences between the ensembles can be clearly visualized. **(B)** Average percent change in RMSE from the Merged when predicting expression levels for each of the top 500 variable genes given the rest of the gene expression data. The standard errors were therefore computed over 500 samples, as opposed to the 100 in panel A.2.

The results are pictured in Figure 4A.1-A.2. The Weighting Trees approach is the overall best for predicting OS. The Merged learner performs several orders of magnitude worse than the rest, in both relative and absolute terms. Conversely, the Unweighted, Weighting Trees, and Weighting Forests methods have low absolute prediction Log Loss. This agrees with the results from simulations using binary outcomes shown in Figure S1 from the supplement.

We also evaluated the performance of the ensembles on a continuous outcome. As there was no continuous clinical variable, we considered the task of predicting gene expression levels. Clinical decisions are often informed by expression levels of certain genes, thus predicting these levels when they may be missing can be useful in determining treatment. We used six studies in this analysis. The sample sizes range from 21 to 195 subjects, with 511 total across studies. The six datasets have 1,312 gene features in common. We predicted the expression level of one gene given the rest of the expression data, for the 500 most variable genes included in the features. Our performance metric was the percent change in average RMSE from the merged learner. The results, averaged across all 500 genes tested with associated 95% confidence intervals, are pictured in Figure 4B. The general ordering the performance of the ensembling approaches follow that of the discrete outcome case, with the Weighting Trees approach outperforming the rest. It is slightly superior to Weighting Forests, and both surpass the Unweighted. All three ensembles significantly improve upon the Merged learner; this slightly differs from the results seen in the simulations, in which the Merged approach typically outperforms the Unweighted. This difference is likely due to different composition of the gene expression data in the breast cancer studies compared to that in the simulations, and provides more motivation for using ensembling over merging when dealing with multiple datasets.

## Discussion

In this paper we proposed and investigated a methodology for building ensembles of trees trained on multiple studies, to achieve robustness to cross-study heterogeneity. Patil and Parmigiani^2^ introduced a general multi-study learning architecture that uses stacking to combine predictions built on separate studies. In this setting, using Random Forests as a single study learner, we compared weighting each forest to form the ensemble, as they did, to extracting the individual trees trained by each Random Forest and weighting them directly. Our methodology thus extends their methods.

Our results broadly indicate that Weighting Trees is often more effective than Weighting Forests, sometimes by a considerable margin. In turn, this suggests that more efficient families of cross-study learners can be constructed by “unpacking” learners that are themselves based on ensembles, and weighting the individual components directly. Our results may depend on the specific implementation of the stacking algorithm used to re-weight the trees. We used ridge regression stacking, but other approaches to regularization would be worth exploring.

Our strategy can be applied whether or not the training sample is naturally divisible into different studies or sub-populations. We investigated this case as a limiting case when heterogeneity is close to 0. The findings from our baseline analysis (see Figure 1) suggest that for larger datasets, it may be advantageous to train SSL’s on subsections and ensemble using stacking weights that reward prediction across different sections of the data, rather than simply training a learner on the whole dataset. Focusing SSL’s on fewer observations may allow the learners to capture more of the specific features of the dataset, which can then be combined in a way that promotes cross-study generalizability. Simply training one learner on the entire dataset may ignore potential heterogeneity in feature distributions between subsets of observations. Further exploration of the single study setting can be found in the supplement.

## Supplementary Material

1

## Figures and Tables

**Table 1:** Tree structure summaries and variable importance measures for each ensembling approach, by tree weight. The left three columns are average tree-level measures. The rightmost column measures the proportion of the total sum of variable importance scores attributed to true variables. Variable importance is computed as follows: for each tree, the prediction error on the out-of-bag portion of the data is recorded (using MSE for regression). Then the same is done after permuting each predictor variable. The difference between the two is then averaged over all trees, and normalized by the standard deviation of the differences.

Tree weight percentile	Average tree weight	Freq. of true variables/tree	# of variables in interactions	Proportion of total varImp by true vars.

Weighting Trees				

90–100%	**0.068**	**0.402**	**1.459**	**0.699**
	(0.0675, 0.0685)	(0.400, 0.404)	(1.443, 1.475)	(0.671, 0.727)
0–10%	**0.001**	**0.143**	**0.48**	**0.502**
	(0.001, 0.001)	(0.141, 0.145)	(0.468, 0.492)	(0.477, 0.527)
0–100%	**0.026**	**0.265**	**0.948**	**0.655**
	(0.0259, 0.0261)	(0.264, 0.266)	(0.937, 0.959)	(0.627, 0.683)

Weighting Forests				

90–100%	**0.0463**	**0.368**	**1.348**	**0.704**
	(0.0458, 0.0468)	(0.364, 0.372)	(1.326, 1.37)	(0.675, 0.733)
0–10%	**0.0091**	**0.171**	**0.571**	**0.512**
	(0.0089, 0.0093)	(0.169, 0.173)	(0.558, 0.584)	(0.486, 0.538)
0–100%	**0.0256**	**0.265**	**0.948**	**0.655**
	(0.0254, 0.0258)	(0.264, 0.266)	(0.937, 0.959)	(0.627, 0.683)

Merged				

0–100%	**0.01**	**0.504**	**1.889**	**0.624**
		(0.502, 0.506)	(1.869, 1.909)	(0.600, 0.648)
