# Supplementary material for: Tree-Weighting for Multi-Study Ensemble Learners
Source: Pac Symp Biocomput. Author manuscript; Available in PMC 2020 Jan 24. (PMC6980320)
Supplement: 1 [file NIHMS1061174-supplement-1.pdf]

Supplement to “Tree-weighting for multi-study ensemble learners”

Maya Ramchandran[1], Prasad Patil[1,2], and Giovanni Parmigiani[1,2]

October 2, 2019

[1] Department of Biostatistics, Harvard T.H. Chan School of Public Health
[2] Department of Data Sciences, Dana-Farber Cancer Institute

This document serves as a supplement to the main article “Tree-weighting for multi-study ensemble learners”. Here, we present two supplementary simulations: the first explores the efficacy of ensembling approaches on a binary outcome, and the second evaluates the effect of increasing the number of trees per forest on performance using a continuous outcome. We also present additional results, including a simulation and a data example, that extend the ensembling paradigms considered in the single study setting.

Binary outcome simulation

Figure S1 displays average percent change in prediction Log Loss from the Merged over 100 iterations for the various ensembling approaches for three different scenarios, when the outcome is binary. Out of 100 total features present, 10 features affect the outcome; the outcome is continuous. Panel A corresponds to interaction scenario (1) as described in the Simulating Datasets section of the main text, while panel B corresponds to scenario (2). Panel C considers no interactions and considers the effect of increasing the level of feature effect heterogeneity. The datasets were generated in the same manner as described in the Simulating Datasets section, with one exception: the generated continuous outcome was dichotomized based on quantile, with outcomes values falling above the 75th percentile being assigned a value of 1, and 0 otherwise. This created a binary outcome for the analysis.

In all three scenarios considered, all three ensembling approaches almost double their improvements over the Merged, with Weighting Trees outperforming the others throughout. In panels A and C, there are clear distinctions in the performances of each approach - interestingly, the Unweighted ensemble surpasses Weighting Forests, which is not a result we see in the continuous outcome setting. In panel B, the Weighting Forests and Unweighted approaches are nearly indistinguishable, while Weighting Forests exceeds them both.

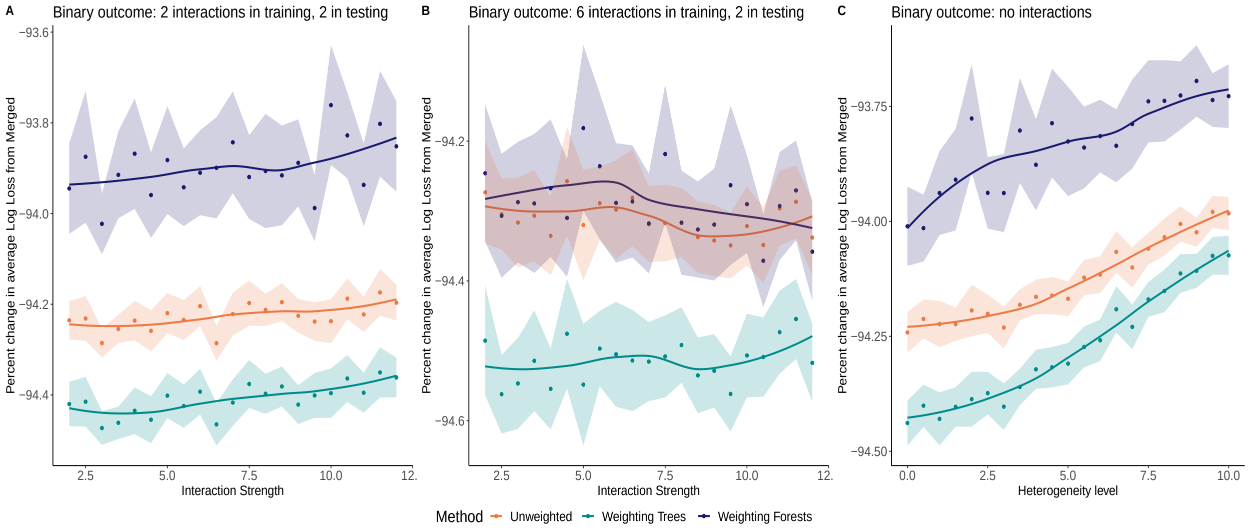

Figure S1: Average percent change in prediction Log Loss from the Merged for each of the ensembling approaches (color labeled) on a binary outcome variable, as a function of increasing interaction strength or heterogeneity. **(A)** 2 datasets with interaction terms between features in the outcome-generating generating mechanism are included in the training set, and 2 are included in the testing set. **(B)** 6 datasets with interactions are included in the training set, 2 in the testing set. **(C)** No datasets with interaction terms are included in either training or testing, and performance is evaluated for increasing feature effect heterogeneity. For all three scenarios, and across parameter levels, Weighting Trees outperforms all other approaches, and all three ensembling approaches vastly outperform the Merged.

Increasing the number of trees in the ensemble

Figure S2 displays Average RMSE's of the ensembling approaches as a function of increasing number of trees per forest in each ensemble. The data was generated in the same manner as described in the Simulating Datasets section of the main text, with 10 datasets used for training and 5 for validation at every iteration. Therefore, if n equals the number of trees per forest, the total number of trees per ensemble is $n \times 10$. We tested ensembles with total number of trees ranging from 100 to 7500, in order to determine how the relationship between the approaches is affected by increasing ensemble size. Due to the great computational time and resources required for training the larger forests, we conducted 20 iterations (instead of the typical 100) at each forest size, resulting in larger confidence bands. We find that overall, the relationship between ensembling approaches mirrors that from Figure 2 of the main text, with Weighting Trees outperforming the others throughout. Furthermore, we see that these patterns remain constant as ensemble size increases.

There is a steep improvement in performance for the smaller ensembles as the number of trees per forest increases, but the performance of all approaches plateaus for larger ensembles; for instance, there does not appear to be significant improvements from using 750 trees per forest over 100 trees per forest. The vast increase in computational time necessary to train larger ensembles does not seem to result in appreciable gains. These findings motivate our choice of using 10 trees per forest in our analyses.

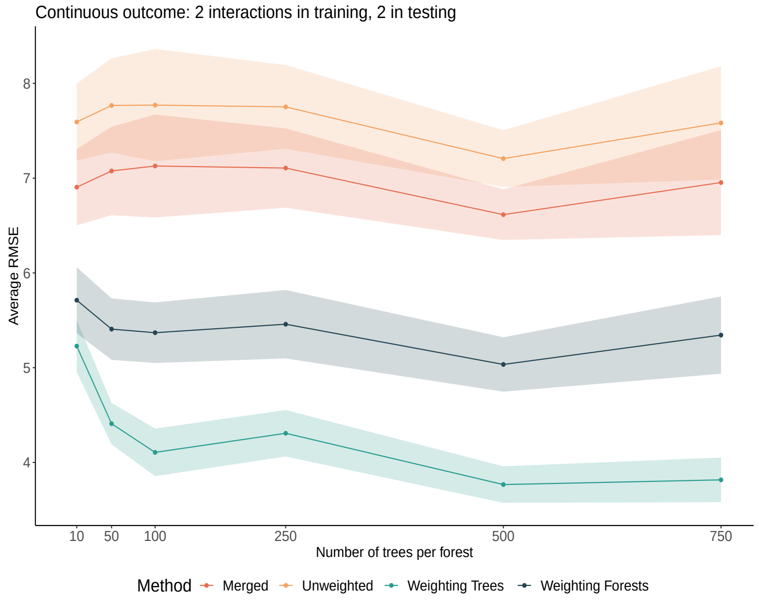

Figure S2: Average RMSE's of ensembling approaches (color labeled) on a continuous outcome variable, as a function of increasing number of trees per forest, over 20 iterations at each level. 2 datasets with interaction terms between features in the outcome-generating generating mechanism are included in the training set, and 2 are included in the testing set. Across all parameter levels, Weighting Trees outperforms all other approaches, and the relationship between approaches is conserved as the number of trees per ensemble increases.

Results for the single study setting

The Combined approach

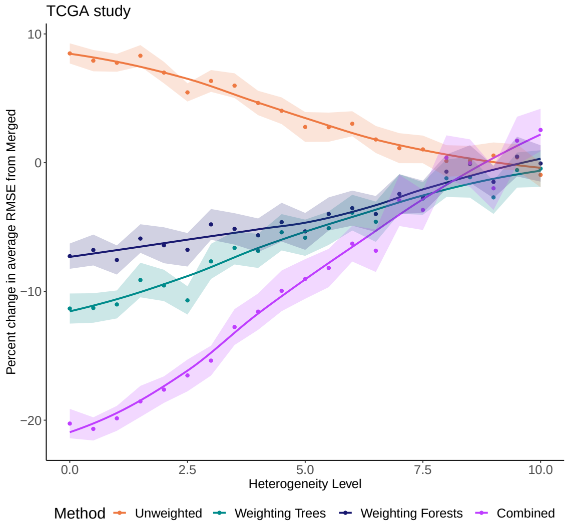

Figure S3: Performance of the Combined approach compared to the other ensembling methods on the TCGA study; the TCGA study is randomly split into 5 sub-datasets at every iteration for training and testing. The Combined significantly improves on the rest for lower levels of heterogeneity between the sub-datasets.

Our simulations so far focused specifically on the comparison between weighting trees and weighting forests, using learning from the merged data as a benchmark. Table 1 from the main text suggests that trees identified by single study learners capture different features compared to trees trained on merged data sets. Having established that weighting individual trees improves prediction ability, we can now ask whether this concept can be extended to collections of trees trained both on single studies and merged data. Table 1 reveals that the Merged approach can more frequently identify true predictive features. It is possible that useful trees may be identified often in the merged training, but may not be adequately leveraged because of equal weighting.
To explore this, we implemented a Combined Weighting Trees method (Combined for short), which computes stacked regression weights with a ridge constraint on the union of the 100 trees trained on the merged dataset and the $10 \times 10 = 100$ trees trained by the SSL approach; the resulting ensemble comprises 200 trees. We then reconsider the simulation setting of Figure 1C of the main text.
Figure S3 summarizes the results. The Combined approach improves upon all of the other ensembles for most heterogeneity levels considered. The greatest gap is observed at 0, when no feature effect heterogeneity is included. This separation in performance decreases with heterogeneity increases. As heterogeneity increases, so does the importance of modeling both the changes in features distribution and relationship between the features and the outcome; this results in a decreasing difference between including trees trained on the Merged in the Combined approach and simply Weighting Trees or Weighting Forests. Subsequently, when tested in the multi-study simulation setting described previously, the Combined approach did not improve upon the weighting trees approach, possibly because of the more central role of feature distribution heterogeneity.

Data application

We next considered the use of ensembling methods on large real single datasets. The MetaGxBreast package from Bioconductor in R provides a collection of Breast Cancer Transcriptomic Datasets that are part of the MetaGxData package compendium [1]. For this analysis, we used the GSE25066 study, which contains 508 subjects, and the Metabric study, which contains 1989 subjects; in both datasets, we included the 100 gene features that exhibit the most variability, as well as any clinical features with complete data. For the GSE25066 study, we looked at two outcomes: DMFS (Distant Metastasis Free Survival) status, and days to DMFS. Days to DMFS describes the number of days from diagnosis to appearance of a distant metastasis. A distant metastasis refers to cancer that has spread from the original (primary) tumor to distant organs or distant lymph nodes. DMFS status is a binary variable that describes whether or not appearance of distant meastasis occurs during the study period. For the Metabric study, we considered the outcome Days to Death, which measures days from diagnosis to death, if death occurs within the study period. Patients within the study with missing values (either due to dropout or because death did not occur during the study period) were omitted from the analysis, yielding 1989 patients with complete data.
When constructing ensembles using the GSE25066 study, we split the dataset into five randomly chosen, equally sized sub-datasets, trained on four, and tested on the fifth. The results for the two outcomes considered for 100 iterations of such splits are shown in Figure S4A-B.

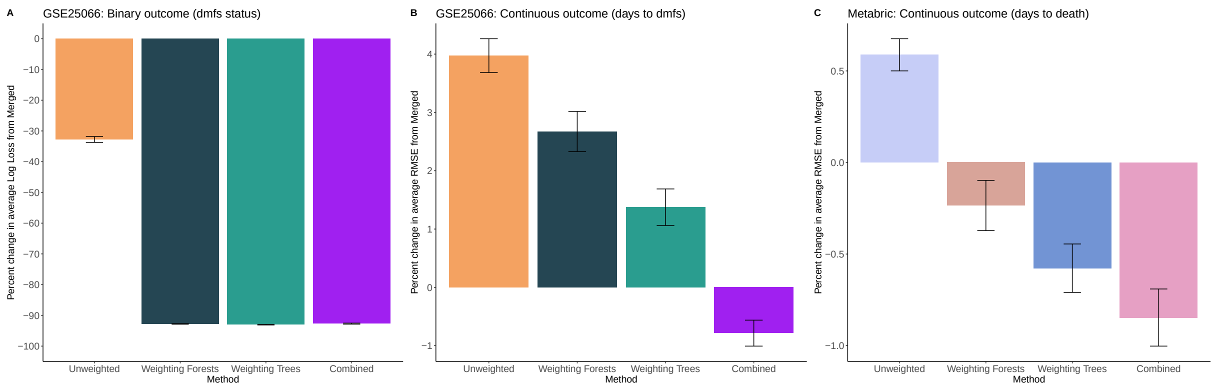

Figure S4: Performance of the different ensembling approaches on the breast cancer datasets in the single study setting, with associated 95% confidence intervals. Confidence intervals for all panels were obtained by iterating through the training and testing process 100 times. **(A)** Average percent change in prediction Log Loss from the Merged for each of the ensembling approaches on the binary outcome variable, Distant Metastasis Free Survival (DMFS) status. For every iteration, the GSE25066 Breast Cancer dataset (508 patients) was randomly split into 5 equally sized sub-datasets; 4 were then randomly chosen for training, and the 5th was used for testing. **(B)** Average percent change in RMSE from the Merged when predicting the continuous outcome variable, days to DMFS, using the GSE25066 Breast Cancer dataset in the same manner as described in (A). **(C)** Average percent change in RMSE from the Merged when predicting the continuous outcome variable, days to death. For every iteration, the Metabric Breast Cancer dataset (1989 patients) was randomly split into 11 equally sized sub-datasets; 10 were then randomly chosen for training, and the 11th was used for testing.

For the Metabric study, we split the dataset into 11 pseudo-datasets, trained on 10, and tested on the 11th. The results for 100 iterations of such splits are shown in Figure S4C.
As evidenced by Figure S4A, all ensembling methods vastly outperform the Merged when predicting a binary outcome in the GSE25066 dataset; the three approaches that use cross-study weights are almost twice as accurate, while the Unweighted has more modest gains. The differences between the three top performers are negligible, indicating that for a binary dataset, using any of the weighting approaches that reward generalizability of trees or forests is sufficient to improve prediction ability by the same amount. The near equality of Weighting Trees and Weighting forests mirror the pattern seen in the simulations shown in Figure 1 of the main text; however, the Combined does not improve upon either, unlike the simulations in Figure S3. This indicates that the SSL's are able to capture enough of the whole dataset structure that the addition of trees trained within the Merged does not significantly improve performance.
Figure S4B shows the results from using the continuous outcome, Days to DMFS, in the GSE25066 dataset. Here, only the Combined approach described in the simulations section yields a better predictor than the Merged, and the improvement itself is only of the order of 1%. All other ensembles perform slightly worse than the Merged, with Weighting Trees producing the smallest difference. Containing 508 observations, the GSE25066 dataset is relatively large compared to many other gene expression and clinical datasets, which can typically be more on the order of 100-200 observations. However, we also wanted to deter-

mine whether the splitting method would be more appropriate on much larger datasets when the aim is to predict a continuous outcome.

To this end, we tested the performance of the ensembles on the Metabric dataset, which contains 1989 observations with non-missing outcome data (days to death); the results are displayed in Figure S4C. Here, all cross-study ensembling approaches slightly outperform the Merged, with the improvements all under 1%. Again, the Combined approach is the best of all methods considered, but unlike in the GSE25066 dataset, the Weighting Trees and Weighting Forests approaches yield marginally better predictions than the Merged. Although the range of improvement is not high, these results suggest that as the dataset gets larger, there is more benefit to using all cross-study ensembling. Overall, regardless of the size of the dataset or the type of outcome, the Combined ensemble always outperforms the Merged; this highlights its utility in the single dataset setting, particularly when dealing with a binary outcome.

The difference between the binary and continuous outcome results for the same dataset indicate the importance of the structure of the feature-outcome response in determining the performance of the ensembling approaches. We saw for the simulations that as the level of heterogeneity increased, all approaches converged; it appears that presence of considerable heterogeneity in the relationship between the features and outcome across the observations in the full dataset negatively impacts the performance of the ensembling approaches when the sub-datasets are chosen randomly. This applies to both datasets analyzed in this section.

Discussion

The results of the single study data example (see Figure S4) suggest that any weighting approach that rewards cross-study generalizability may be used in constructing ensembles for use on datasets with a binary outcome that are large enough to reasonably split into sub-datasets, as they vastly outperform a single learner trained on the entire dataset. Further work should be done in developing methods to determine advantageous groupings of observations based on sources of heterogeneity in the joint distribution of the predictive features. Since there are only two levels within a binary outcome, nuanced differences in the feature-outcome relationship across observations are harder to detect, as the amount of heterogeneity that maps to a particular label is significantly larger than that which would map to a particular continuous outcome value. This may be the reason for the substantial increase in performance of the weighted ensembles over the Merged even when the sub-datasets are randomly chosen.

For a continuous outcome, an important distinction to make between the simulations and data applications is that in the simulations, the outcome is defined to be linearly related to the features, while we don't know whether this is true in the data applications. In the single-study setting, creating pseudo-studies generates simpler trees, which may be either advantageous or disadvantageous depending upon the complexity of the relationship between the covariates and outcome. In the simulations, in which this relationship is very clearly defined, generating simpler trees produces significant improvements in ensemble predictions. However, we see more ambiguous results in the continuous outcome single study data examples (Figure S4, B-C); one possibility is that in this case, the covariate-outcome relationship

is too complex to afford the advantages of simpler trees, resulting in only marginal gains in ensembling over merging.
Trees identified by single study learners capture different features compared to trees trained on merged data sets. We explored whether larger collections of trees including those trained both on single studies and merged data would provide improvements. This combined approach is advantageous when heterogeneity in the joint distribution of the features is low, particularly in the single study setting. Interestingly, some of these gains persist when there is moderate heterogeneity in the relationship between features and outcomes. This is intriguing and deserves further study, as the importance of modeling effect heterogeneity is paramount, and trees that do not take this into account are unlikely to promote generalizability to new datasets. Overall, in the single study setting, the addition of trees that do not model sub-study heterogeneity and take into account the structure of the entire dataset enhances the performance of the trees within the SSL's when no external between-study heterogeneity is added to the sub-studies. This suggests that the combined approach is a valuable candidate when considering ensemble methods for large single datasets.

References

[1] D. M. A. Gendoo, M. Zon, V. Sandhu, V. S. K. Manem, N. Ratanasirigulchai, G. M. Chen, L. Waldron and B. Haibe-Kains, Metagxdata: Clinically annotated breast, ovarian and pancreatic cancer datasets and their use in generating a multi-cancer gene signature, *Scientific Reports* **9**, p. 8770 (2019).
